# Induction Chemotherapy Improved Long Term Outcomes in Stage IV Locoregional Advanced Nasopharyngeal Carcinoma

**DOI:** 10.7150/ijms.42005

**Published:** 2020-02-10

**Authors:** Yu-Wen Wang, Sheng-Yow Ho, Sung-Wei Lee, Chia-Chun Chen, Shieh Litsu, Wen-Tsung Huang, Ching-Chieh Yang, Chia-Hui Lin, Hsuan-Yu Chen, Li-Ching Lin

**Affiliations:** 1 Department of Radiation Oncology, Chi Mei Medical Center, Liouying, Tainan, Taiwan; 2 Division of Hematology-Oncology, Chi Mei Medical Center, Liouying, Tainan, Taiwan; 3 Department of Radiation Oncology, Chi Mei Medical Center, Tainan, Taiwan; 4 Institute of Statistical Science, Academia Sinica, Taipei, Taiwan

**Keywords:** nasopharyngeal carcinoma, locally advanced, induction chemotherapy, concurrent radiotherapy

## Abstract

**Purpose**: We aimed to determine whether adding induction chemotherapy (IC) to concurrent chemoradiation (CCRT) improved outcomes in each stage of locally advanced nasopharyngeal carcinoma (LANPC).

**Methods**: From 2007 to 2013, we retrospectively collected 259 histopathologically identified adult LANPC patients from two campuses in south Taiwan. Among the 238 eligibly treated cases, 156 patients received CCRT (CCRT group) upfront and 82 received IC followed by CCRT (IC group). Of these patients, 130 were stage III (92 patients that received CCRT and 38 that received IC adding CCRT) and 108 were stage IV (76 CCRT and 32 IC adding CCRT). Most chemotherapy regimens for IC are composed of cisplatin (P), 5-fluorouracil (F), and ifosfamide (I), while concurrent chemotherapy (CC) was essentially cisplatin-based. For CCRT as the upfront treatment, a P or PF regimen was usually used in CC. Survival outcomes were accessed with a Kaplan-Meier estimate and a p-value by log-rank test to compare the survival distributions of IC added to CCRT or CCRT as the upfront treatment in all LANPC stage III and LANPC IV patients. The failure free survival (FFS), overall survival (OS), local relapse free survival (LRFS), regional recurrence-free survival (RRFS), distant metastasis-free survival (DMFS), first failure site, and other prognostic factors were analyzed.

**Results**: The median follow-up time of all treated LANPC patients was 59 months. For all LANPC patients, there was a significant difference only in the DMFS favoring IC group (91.5% vs 79.4%, p=0.013). In the subgroup study, for the stage III group, there was no significant difference between the groups for overall OS (IC group 71.3% vs CCRT group 78.7%), FFS (71.5% vs 62.4%) and RRFS (91.9% vs 90.9%). However, inferior LRLS (71.7% vs 91.5%; p = 0.03) was noted for the IC group. In contrast, for stage IV, there were significantly longer OS (75.8% vs 52.6%), FFS (66.8% vs 46.8%), and DMFS (86.0% vs 69.6%; p = 0.02, p = 0.04, and p = 0.03, respectively) rates in the IC group.

**Conclusion**: Adding PIF-based IC to CCRT for the LANPC patients resulted in better outcomes for stage IV patients, but not for stage III patients. A future properly designed study should stratify enough LANPC cases under the structure of the AJCC stage grouping system to determine which subgroups truly benefit from adding IC to CCRT.

## Introduction

Based on the findings of the Intergroup study 1, concurrent chemoradiation (CCRT) has been accepted as the backbone of standard treatment for locoregional advanced nasopharyngeal carcinoma (LANPC) and was verified by meta-analysis and subsequent phase II and III randomized studies, which demonstrated that it has better overall survival (OS) and failure-free survival (FFS) than radiotherapy (RT) alone 2-6. While the toxicity of systemic therapy after CCRT remained a pertinent problem 7, 8, induction chemotherapy (IC) before CCRT (IC + CCRT) 9 is gaining popularity worldwide. Docetaxel (T), cisplatin (P), and 5-fluorouracil (F) (TPF regimen) IC + CCRT method has been shown to improve 3-year FFS, OS, and distant metastasis-free survival (DMFS) when compared with the CCRT alone method in LANPC 10. Their 5-year outcome results confirmed the conclusion in the same groups of patients 11. Recently, gemcitabine (G) and P IC was shown to be as efficient as TPF in treating LANPC in a randomized study 12. This demonstrated adding a non-taxane containing IC regimen could also be superior to CCRT. The NCCN panel voted to change the category of IC + CCRT to 2A from category 3 since 2018 13 because currently available evidence has shown trends toward IC + CCRT being superior compared to CCRT 14. Current NCCN guidelines prefer stage II to IVB patients be enrolled in clinical trials because not all reviews have indicated consistent benefits from adding IC to CCRT in these patients 15. The more advanced the stage, the greater the risk of occult distant metastasis (DM) 16, 17. In addition, acute adverse events and low grade chronic peripheral neuropathy have been found to be drawbacks of adding IC to CCRT 11, 12. Some authors have also highlighted the importance of selecting the right subgroups for IC addition 18, 19. To our knowledge, there are still no randomized studies that have exclusively focused on this issue in subgroups categorized as 2010 AJCC stage III and IV regarding the addition of IC or not for CCRT of LAPNC. The purpose of this study was to explore the role of using a non-taxane containing regimen as the induction treatment for stage III and IV LANPC, respectively, compared to CCRT as the upfront treatment.

## Materials and methods

### Study population

We retrospectively enrolled LANPC (stage III, IVA, and IVB by the AJCC 7th version 2010) patients who were pathologically confirmed and previously untreated patients from our two campuses in south Taiwan. Treatment decisions were made by the lead physicians and most patients were reviewed and monitored by institutional tumor teams consisting of otolaryngologists, medical oncologists, dentists, diagnostic radiologists, nuclear medicine physicians, and radiation oncologists. The Institutional Review Board of the Chi Mei Medical Center approved of the study and the approval number was 10607-L04. Although consent from the enrolled patients was not obtained for this retrospective review, all information was anonymized and de-identified before its analysis.

Pretreatment evaluation for eligible subjects included physical examinations, fiberoptic endoscopy, contrast-enhanced computed tomography (CT) and/or magnetic resonance imaging (MRI) of the nasopharynx covering the region from the skull base to the clavicle, chest x-ray, and abdomen ultrasonography. Positron emission tomography/computed tomography (PET/CT) was optional and a radionuclide bone scan was arranged for those without a PET/CT.

The intention to treat was used to classify the patients' treatments. However, patients with radiotherapy (RT) under 63 Gy and an overall treatment period exceeding 66 days for 74 Gy or 60 days for 70 Gy were regarded as having incomplete treatment and were excluded from the current study. Other exclusion criteria included: 1) recurrent nasopharyngeal carcinoma (NPC), or NPC not being the first diagnosed cancer of the patient; 2) not pathologically proven or no tissue evidence from the nasopharynx locally; 3) incomplete stage work-up prior to treatment; 4) distant metastasis (stage IVC); 5) no concurrent chemotherapy during the RT course; and 6) the patient was underage. The study was approved by the common Institutional Review Board of the two campuses of the Chi-Mei Medical Center.

The induction regimen mainly consisted of PF and ifosfamide (I). The chemotherapy regimen for CCRT was P or PF. Chemotherapy modification was done at the discretion of the primary treating medical oncologists when patients experienced obvious (for example, grade 3) toxicity.

### Radiotherapy

All patients received Intensity Modulated Radiotherapy (IMRT) or Volumetric Arc Therapy with an accelerator or TomoTherapy equipped at the campuses of our center. Inverse planning software was utilized. The gross target volume (GTV) covered the nasopharyneal tumor mass and gross lymphadenopathy. The high risk CTV included the skull base or intracranial area near the nasopharynx and minimally suspected nodes plus the risky neck levels proximal to the gross tumor lesions. The low risk CTV included other lower and more distal risk lymphatic regions for occult micrometastases. Planning target volumes (PTVs) were created by automated expansion of 3 to 5 mm of all GTVs and CTVs to account for any setup error. Normal organs, including the parotid glands, spinal cord, brain stem, optic nerves, ears, optic chiasm, and dysphagia-aspiration related structures were also contoured on the treatment plan. Generally, the prescribed RT doses were around 70~72 Gy to the PTV of the gross tumor volumes of the nasopharynx and positive neck lymph nodes, around 60 Gy PTV of the first clinical tumor volume, and around 45~54 Gy for PTV of the second clinical target volume conventional fractions with sizes from 1.8~2.12 Gy.

### Assessment and follow-up

All patients were encouraged to have regular follow-ups with otolaryngologists, medical oncologists, and radiation oncologists at the outpatient clinic. The patients received regular clinical examinations every 1-3 months in the first year following the completion of therapy, every three months during the second and third years, and then at longer intervals thereafter. Follow-up MRIs were performed before RT for the IC group, regular follow-up MRI or CT scans for all patients every 3-6 months after completing RT, and then every 6-12 months thereafter if no gross tumor recurrence was noted clinically.

### Statistical analysis

A χ2 test or independent t test (or Fisher's exact test for small cell sizes) were used to compare the differences between groups for categorical or continuous variables, respectively. We recorded the demographic data, including gender and age, histology type, and stage. The chemotherapy regimen and given cycles, as well as the radiotherapy dose/fractionation of patients, were also collected. Observed endpoint data included OS, failure free survival (FFS), local relapse free survival (LRFS), regional recurrence-free survival (RRFS), and DMFS. The duration of all survival rates was measured from the end of RT until death or the date of the last follow-up. The first relapse site over the skull base, intracranium, or nasopharynx was deemed to be local, while a retropharyngeal or cervical node above the clavicles was regarded as regional. To compare the result distributions of the IC + CCRT group or upfront CCRT group in the entire LANPC cohort and respective stage III and IV patients, we used the Kaplan-Meier method to estimate the survival curves, and differences between the curves were compared with log-rank tests. A Cox proportional hazard regression model was used to calculate the hazard ratio (HR) and the corresponding 95% confidence interval (CI). All tests were two-sided and P values <0.05 were considered significant. All statistical analyses were carried out using the SPSS statistical program (SPSS for Windows, release 1X.0; SPSS, Chicago, IL, USA) or SAS 9.4 (Cary, NC, USA).

## Results

### Patients and compliance

The medical records of 495 consecutive NPC patients from two campuses of the Chi-Mei medical center were reviewed with a total of 259 adult LANPC patients being enrolled from 2007 to 2013. In the IC + CCRT group, 13 patients were excluded from our analysis [did not receive RT: n = 5; refused further treatment: n = 4; other adverse event (AE)-related non-compliance: n = 4)] While in the CCRT group, 7 patients (refused treatment: n = 2, AE-related non-compliance: n = 5) were excluded from the last analysis.

### Treatment toxicity and dose

A total of 238 treated LANPC cases were eligible. For the IC + CCRT group, during the treatment course, 34 of 82 patients had grade 3 or more AE by definition of the Common Terminology Criteria for Adverse Events and 6 of 82 patients with grade 4 AE. In contrast, for the CCRT group, during their treatment course, there were 82 of 156 patients with grade 3 or more AE, including 4 of 156 patients with grade 4 AE (p = 0.40 and p = 0.07 for grade 3 and 4 AE, respectively). These AE are listed in the supplementary material (Table S1 and Table S2).

Of the IC + CCRT group with documented data from over 75 of 82 patients, the average for each cycle and the accumulated personal mean dose in the induction PIF regimen was 60.3 mg/m^2^ and 175.2 mg/m^2^ for P and over 2.9 cycles and 2688.7 mg/m^2^ and 7617.9 mg/m^2^ for F over 2.8 cycles, respectively. The details of the dose records for P and F are summarized in the supplementary material (Table S3). There was no obvious difference for the accumulated mean dose between stage III (P: 171.0 mg/m^2^, F: 7847.3 mg/m^2^) and stage IV (P: 182.1 mg/m^2^, F: 7810.7 mg/m^2^) cases.

### Characteristics of eligible patients

A total of 156 patients received upfront CCRT and 82 received IC followed by CCRT. The demographic characteristics of the patients are shown in Table 1. The clinical characteristics and demographics were well-balanced in each group, except there were more patients with advanced nodal disease (N2-N3; 86.6% vs 74.4%, p = 0.029) and a trend toward a more advanced stage (stage IVA-IVB) for those enrolled in the IC + CCRT group (53.7% vs 41%, p = 0.063).

Among these 238 eligible patients, 130 were stage III (92 patients received CCRT and 38 received IC + CCRT) and 108 were stage IV (76 CCRT and 32 IC + CCRT). The median follow-up time of all LANPC patients was 59 months (range: 1 to 141 months). For stage III patients, after a median follow-up of 69 months (range: 1 to 141 months), 14 (10.8%), 9 (6.9 %) and 12 (9.2%) patients developed local, regional, and distant failure as the first failure site, respectively. For non-recurrent subjects, the median follow-up time was 74 months (range: 1 to 134 months). For stage IVA~B patients, after a median follow-up of 43 months (range: 3 to 138 months), 18 (16.7 %), 7 (6.5 %) and 19 (17.6%) patients developed local, regional, and distant failure as the first failure site, respectively. For non-recurrent subjects, the median follow-up time was 56 months (range: 9 to 138 months).

The frequency, site of first recurrence and the first salvage treatment modality for local and regional recurrences in each treatment group are summarized in Table 2 and Table 3 for stage III and stage IVA/IVB patients, respectively. Of note, compared with the CCRT group, the local and regional relapse rates appeared higher for the IC + CCRT group (31.6% vs 12.0%) for stage III patients.

### Efficacy

**Fig 1:**
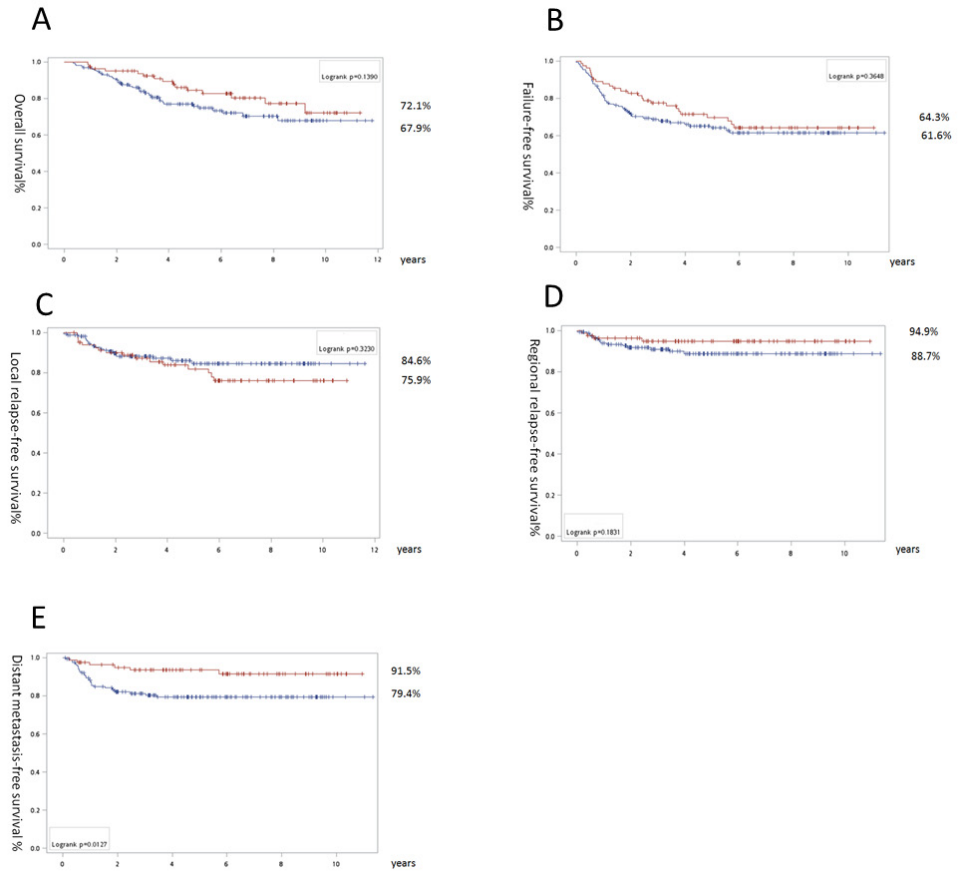
Survival results of all locally advanced nasopharyngeal carcinoma patients: Overall survival (A), recurrence-free survival (B), local relapse free survival (C), regional relapse free survival (D), and distant metastasis-free survival (E). The surviving curves with concurrent chemoradiation as upfront treatment are labeled with a blue line and induction chemotherapy is signified by a red line. The numbers with the percentages indicate the corresponding lines near the final survival rates exceeding follow-up for 11 years.

For all LANPC patients, slight trends toward the IC + CCRT group were noted for OS (72.1% vs 67.9%, p=0.14) and RRFS (94.9% vs 88.7%, p=0.18). However, there was a significant difference in DMFS favoring IC (91.5% vs 79.4%, p=0.013; Fig. 1).

In a univariate cox model of regression, we found stage IV (especially IVB), T4, and old age were associated with a shorter OS. Similarly, for FFS, the significant detrimental factors included stage IV (especially IVA) and T4 (Table 4). For LRFS, stage IVA, T3, and T4 with comorbidity, there were significant parameters for an inferior outcome. Upfront CCRT, stage IV (A or B), and N3 were related to a poorer DMFS (Table 5). However, after multivariate analysis, only stage IV maintained significance (HR = 2.3, 95% CI: 1.31-4.05, p =0.004; HR = 1.69, 95% CI: 1.08-2.66, p =0.022, and HR = 3.09, 95% CI: 1.51-6.33, p = 0.002 for OS, FFS, and DMFS, respectively), while adding induction chemotherapy maintained significance for superior DMFS (HR = 0.3, 95% CI: 0.12-0.74, p = 0.009; Table 6). No other clinical parameters, such as gender, age, comorbidity, and histology classification, had significant influence on clinical outcomes.

**Fig 2:**
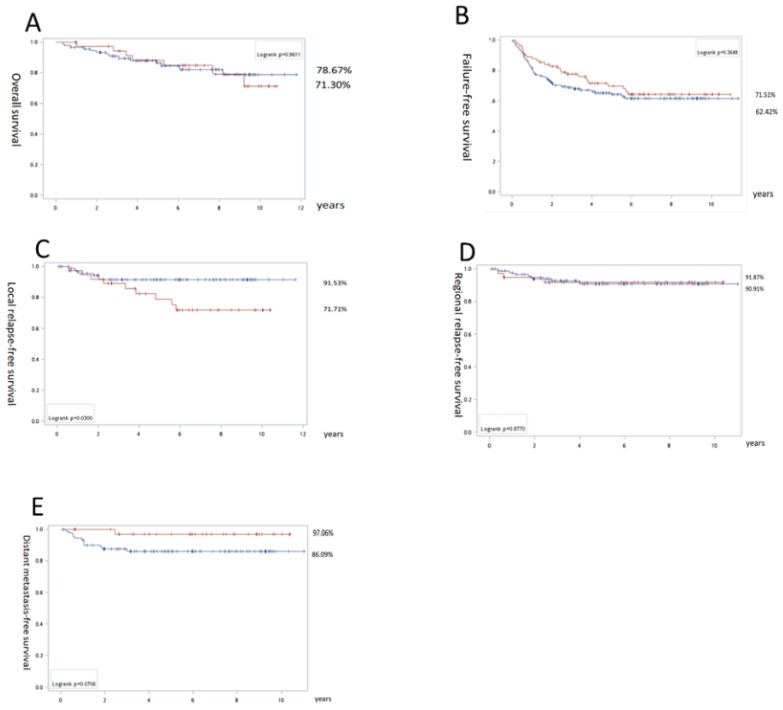
Survival results for stage III nasopharyngeal carcinoma patients: Overall survival (A), failure free survival (B), local relapse free survival (C), regional relapse free survival (D), and distant metastasis-free survival (E). The surviving curves with concurrent chemoradiation as upfront treatment are labeled with a blue line and induction chemotherapy is indicated by the red line. The numbers with the percentages indicate the corresponding lines nearby the final survival rates exceeding follow-up for 11 years.

**Fig 3:**
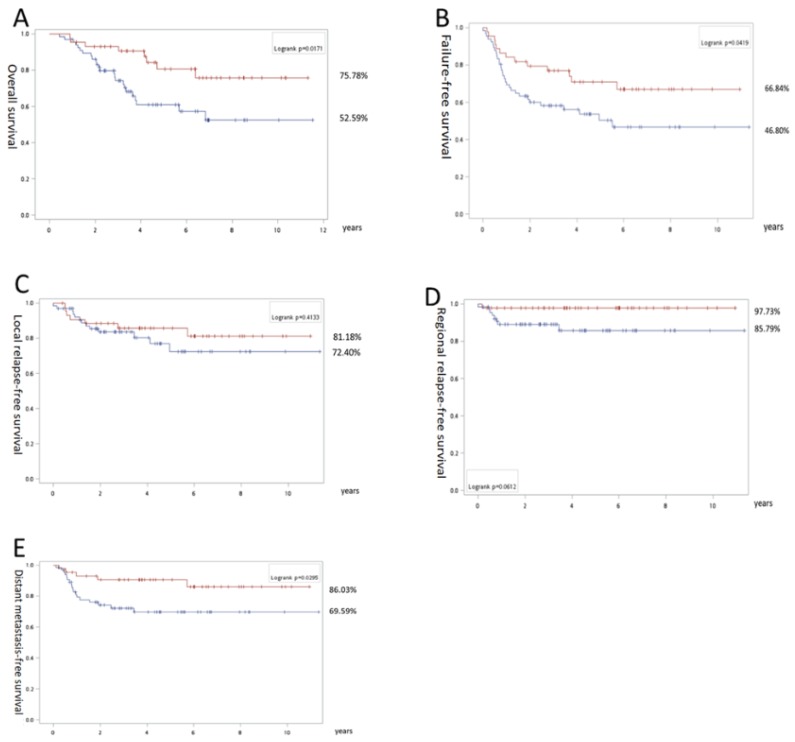
Survival results of stage IV nasopharyngeal carcinoma patients: Overall survival (A), recurrence-free survival (B), local relapse free survival (C), regional relapse free survival (D), and distant metastasis-free survival (E). The surviving curves with concurrent chemoradiation as upfront treatment are labeled with a blue line and induction chemotherapy by a red line. The numbers with the percentages indicate the corresponding lines near the final survival rates exceeding follow-up for 11 years.

In the subgroup study, for stage III patients, there was no significant difference between both groups in terms of OS (IC + CCRT 71.3% vs CCRT 78.7%), FFS (71.5% vs 62.4%) and RRFS (91.9% vs 90.9%; Fig. 2). However, inferior LRLS (71.7% vs 91.5%; p = 0.03) was noted for the IC+ CCRT group. In addition, there was a trend favoring DMFS (97.1% vs 86.1%; p = 0.07) for the IC + CCRT vs CCRT groups. In contrast, for stage IV, we found significantly better OS (75.8% vs 52.6%), FFS (66.8% vs 46.8%), and DMFS (86.0% vs 69.6%) with p = 0.02, p = 0.04, and p = 0.03, respectively, and the trend favoring RRFS (97.7% vs 85.8%; p = 0.06) in the IC + CCRT group (Fig. 3).

## Discussion

If we count the entire LANPC cohort, the overall results of the OS and FFS rates were similar to other published series 11, 12 with better DMFS in the IC + CCRT group. However, there were distinct results if the outcomes for the stage III and stage IV LANPC cases were calculated separately. In stage III patients, there were no major survival outcome differences, except LRFS was superior in the CCRT group. Yet there were remarkably different survival outcomes in stage IV cases favoring the IC + CCRT group.

These survival differences were supposedly mainly due to the differences in risk among the occult DM for each stage, which was considered as the major portion of subsequent cancer specific mortality. There were also some randomized clinical trials, systematic reviews and a meta-analysis report that favored IC as the upfront treatment for LANPC 11, 12, 14. A reduction in the incidence of occult DM recurrences by IC was a potential explanation for their survival advantage. We believed there may be major heterogeneity in the prognosis spectrum among the LANPC patients. Stage III patients are considered to have much better cancer specific survival than Stage IV patients 16, 17. It is therefore important to select a subgroup of people with LANPC (stage III-IVB) to determine the true benefit of adding IC to CCRT. We know N3 (a condition of stage IVB) is almost a surrogate of occult DM 12 and N2 (one condition of stage III) was also related to the high risk of DM but to a lesser extent than N3 20. Note, some situations in T4 (a condition of stage IVA), such as cavernous sinus invasion, was associated with an elevated risk for occult DM 17, 21. Thus we think the overall risk for occult DM in stage III patients is reasonably lower than that in the stage IV cases. Thus it seems reasonable to suggest adding IC to CCRT did not lead to survival benefits in the stage III NPC patients. There was no significant statistical difference in OS and FFS, and even the trend of DMFS (p = 0.07) toward the IC + CCRT group was completely offset by the inferiority of their LRFS (p = 0.03). There was a higher late local relapse rate for stage III patients that underwent IC + CCRT that often happened 2-3 years after treatment, and it was considered to be at least partially responsible for the missing superiority of IC + CCRT. We found in our T3 cases, there were 17 (44.7%) more in the IC + CCRT group than in the CCRT group, which had 26 (28.3%, p = 0.257). This might be related to the poorer local control in stage III patients in the IC + CCRT group. However, the exact reason for a higher local relapse rate only occurring in stage III, but not in stage IV LANPC, remains unclear. There have been no reports regarding stage III in the literature that discuss adding IC to CCRT, and this therefore demands further investigation with a proper study design.

Wu reported a similar result with the entire LANPC group and IC + CCRT was associated with even poorer local control outcomes 19. They recruited 90 LANPC patients for CCRT (66 stage IIB-III, 24 stage IVA-B) and 38 patients for IC + CCRT (17 IIB-III, 21 IVA-IVB). There were similarities between Wu's study and our study. First, both were retrospective observational studies carried out in an endemic area. They collected nearly 95% of the WHO type II-III histology types of the NPC cases in southern Taiwan. Second, the standard of AJCC staging in LANPC was identical. Third, two studies used a non-taxane cisplatin and fluorouracil-based regimen, although some details differed. Fourth, the median follow-up period was comparable (59 vs 53 months). We had a slightly longer follow-up period, even though we measured it from the end of the RT date. The differences are listed below. First, we included more cases (238 vs 128), making survival analysis for each subgroup feasible. Second, we had more positive findings for OS, FFS, and DMFS of stage IV LANPC patients favoring the IC + CCRT group. This finding was similar to important publications regarding the IC + CCRT issue 11, 12, 14. There was only one randomized trial reported in the literature that enrolled Taiwanese patients and investigated IC + CCRT versus CCRT and stage IV LANPC issue. Their median follow-up period was six years. Mitomycin C, epirubicin, and leucovorin were added to cisplatin and fluorouracil as the IC regimen 22. They found only a difference in FFS between the IC + CCRT and CCRT groups. The author believed the myelotoxicity of mitomycin C was too strong and the subsequent dose of cisplatin and RT was significantly limited, which was responsible for the failure to identify the superiority of IC + CCRT in OS.

One limitation of this study was we reported the long-term follow-up outcomes without reporting the post-treatment responses because the aim of this study was to determine the relative long-term efficacy of IC + CCRT and CCRT and we did not analyze the initial response. In addition, some of the records regarding the initial responses were missing. We also did not report treatment-related toxicities after their treatment in this retrospective study because the clinicians only tended to record the major complications related to their treatments instead of recording all side effects as needed in a clinical trial. This could have resulted in underestimation of minor but still significant adverse effects. Since this was a retrospective report, we did not measure the effect of biomarkers, such as the Epstein-Barr virus (EBV) DNA level 23 and plasma lactate dehydrogenase (LDH) level 24. We only included NPC cases that completed their RT course in the proper short overall treatment time with a high dose as being eligible for subsequent analysis because we thought only such cases would have radical treatments. This claim was considered to be compatible with the work of Zhang et al. Their result of intention to treat and per-protocol was the same 12. Completing RT in a short time with a high RT dose highlighted the treatment intensity as being high and also the superiority of IC + CCRT in stage IV LANPC was made clear. This consideration also echoed the suggestion of Li that there was more treatment intensification for extremely high risk subgroups 11. Thus, we consider it promising to resolve the issue by collecting enough cases and stratifying them under the structure of the AJCC staging system to seek the best choice for subgroups to adopt IC + CCRT or CCRT as upfront treatment under a properly designed study.

## Conclusion

Adding PIF-based IC to CCRT for LANPC patients resulted in better outcomes in stage IV but not stage III patients. A further properly designed study should stratify enough LANPC cases under the structure of the AJCC staging system to determine which subgroups truly benefit from adding IC to CCRT.

## Supplementary Material

Supplementary tables.

## Figures and Tables

**Table 1:** Eligible patient demographics and disease characteristics

	IC + CCRT (n = 82)		CCRT (n = 156)		p value
Characteristics	No.	%	No.	%	
Age (years)					0.568
< =50	52	63.4	93	59.6	
> 50	30	36.6	63	40.4	
Gender					0.292
Male	54	65.9	113	72.4	
Female	28	34.1	43	27.6	
Pathologic feature					0.396
WHO Type I	0	0	3	1.9	
WHO Type II~III	77	93.9	146	93.6	
N/A	5	6.1	7	4.4	
AJCC 2010 stage					0.025
III	38	46.3	92	59	
T3 in stage III	17	44.7	26	28.3	0.257
IV	44	53.7	64	41	*0.063
IVA	20	24.4	41	26.3	
IVB	24	29.3	23	14.7	
T1~T2	31	37.8	74	47.4	0.155
T3~T4	51	62.2	82	52.6	
N0~N1	11	13.4	40	25.6	0.029
N2~N3	71	86.6	116	74.4	

IC + CCRT: induction chemotherapy + concurrent chemoradiation; CCRT: concurrent chemoradiation; N/A: not available from the documentation; AJCC: American Joint Committee on Cancer; * if stage III versus stage IV; WHO: World Health Organization.

**Table 2:** Frequency, site of first recurrence, and first salvage treatment modality for 2010 AJCC stage III local and regional recurrences

	IC + CCRT group		CCRT group	
	(n = 38)		(n = 92)	
	No.	%	No.	%
First failure site				
Local	9	23.7	5	5.4
Regional	3	7.9	6	6.5
Distant	1	2.6	11	12.0
First treatment modality for local and regional relapse				
Surgery ± CTx	1	8.3	3	27.3
RT ± CTx	9	75	6	54.5
CTx	1	8.3	1	9.1
Nil	1	8.3	1	9.1

IC + CCRT: induction chemotherapy + concurrent chemoradiation; CCRT: concurrent chemoradiation; Ctx: chemotherapy; RT: radiotherapy.

**Table 3:** Frequency, site of first recurrence, and first salvage treatment modality for 2010 AJCC stage IVA ~ IVB local and regional recurrences

	IC group		CCRT group	
	(n = 44)		(n = 64)	
	No.	%	No.	%
First failure site				
Local	6	13.6	12	18.8
Regional	1	2.3	6^a,b, c^	9.4
Distant	4	9.1	15	23
First treatment modality for locoregional relapse				
Surgery ± Ctx	2	28.6	2	11.8
RT ± Ctx	2	28.6	10	58.8
CTx	3	42.9	1	5.9
Nil	0		2	11.8

IC + CCRT: induction chemotherapy + concurrent chemoradiation; CCRT: concurrent chemoradiation; ^a^One patient had regional recurrence and mediastinal metastases concurrently and the other had regional and lung metastases simultaneously; ^b^One patient had regional recurrence and lung metastases concurrently; ^c^One patient had local and regional relapse and liver metastasis concurrently; Ctx: chemotherapy; RT: radiotherapy.

**Table 4:** Univariate Cox regression analysis for overall survival and failure free survival

	Overall survival				Failure free survival		
	HR	95% CI	*p*-value		HR	95% CI	*p*-value
IC (reference: CCRT)	0.77	0.57-1.04	0.091		0.8	0.5-1.27	0.346
Stage IVA (reference: III)	2.05	0.99-4.24	0.052		1.76	1.07-2.9	0.027
Stage IVB (reference: III)	2.94	1.16-7.47	0.023		1.44	0.81-2.55	0.214
Stage IV (reference: III)	2.35	1.28-4.33	0.006		1.76	1.06-2.9	0.027
T3 (reference: T1-T2)	1.44	0.67-3.11	0.348		1.12	0.61-2.07	0.709
T4 (reference: T1-T2)	2.58	1.38-4.83	0.003		1.91	1.17-3.11	0.01
T4 (reference: T3)	1.84	0.91-3.72	0.092		1.76	0.98-3.17	0.061
N2 (reference: N0-1)	0.51	0.26-1	0.051		0.7	0.41-1.2	0.195
N3 (reference: N0-1)	0.91	0.71-1.18	0.488		0.9	0.47-1.73	0.759
N3 (reference: N2)	1.65	0.83-3.29	0.157		1.31	0.75-2.29	0.35
WHO classification type 1 (reference: 2)	1.18	0.16-8.57	0.873		0.91	0.13-6.53	0.923
Female (reference: male)	0.59	0.3-1.14	0.118		0.69	0.41-1.16	0.162
Age ≥ 50 (reference: < 50)	1.75	1.02-3.01	0.044		1.38	0.89-2.13	0.152
With comorbidity* (reference: no)	1.63	0.95-2.81	0.078		1.45	0.93-2.26	0.098

IC: induction chemotherapy + concurrent chemoradiation; CCRT: concurrent chemoradiation; HR: hazard ratio; CI: confidence interval; WHO: World Health Organization; *comorbidity: common comorbidities include chronic diseases involving the heart, vessels, brain, lung, liver, kidneys, or the immune system and metabolism such as hypertension and diabetes.

**Table 5:** Univariate Cox regression analysis for local relapse free survival and distant metastasis-free survival free survival

	Local relapse free survival				Distant metastasis-free survival		
	HR	95% CI	p-value		HR	95% CI	p-value
IC (reference: CCRT)	1.38	0.71-2.66	0.342		0.35	0.15-0.85	0.02
Stage IVA (reference: III)	2.36	1.15-4.86	0.019		2.22	1.07-4.6	0.032
Stage IVB (reference: III)	1.19	0.47-3.06	0.713		2.34	1.18-4.62	0.015
Stage IV (reference: III)	1.82	0.94-3.51	0.077		2.58	1.28-5.18	0.008
T3 (reference: T1-T2)	2.73	1.02-7.34	0.046		1.02	0.72-1.44	0.93
T4 (reference: T1-T2)	4.64	1.96-10.99	0.001		1.13	0.92-1.39	0.259
T4 (reference: T3)	1.74	0.79-3.82	0.17		1.56	0.53-4.57	0.416
N2 (reference: N0-1)	0.54	0.25-1.16	0.112		0.81	0.51-1.28	0.367
N3 (reference: N0-1)	0.52	0.19-1.44	0.209		1.14	0.84-1.54	0.409
N3 (reference: N2)	0.99	0.4-2.47	0.98		2.33	1.01-5.37	0.048
WHO classification type 1 (reference: 2)	N/A.	N/A	0.989		0.47	0.06-3.42	0.454
Female (reference: male)	0.96	0.46-2.01	0.918		0.57	0.25-1.29	0.176
Age ≥ 50 (reference: < 50)	1.79	0.93-3.47	0.083		1.13	0.58-2.19	0.728
With comorbidity* (reference: no)	2.09	1.09-4.03	0.027		1.34	0.68-2.63	0.402

IC: induction chemotherapy + concurrent chemoradiation; CCRT: concurrent chemoradiation; HR: hazard ratio; CI: confidence interval; WHO: World Health Organization; N/A: not applicable due to great disparity between the two groups; *comorbidity: common comorbidities include chronic diseases involving the heart, vessels, brain, lung, liver, kidneys, or immune system and metabolism such as hypertension and diabetes.

**Table 6:** Multivariate Cox regression analysis for local relapse free survival and distant metastasis-free survival free survival

	Overall survival				Failure free survival				Local relapse free survival				Distant metastasis-free survival		
	HR	95% CI	p-value		HR	95% CI	p-value		HR	95% CI	p-value		HR	95% CI	p-value
IC (reference: CCRT)	0.58	0.31-1.07	0.081		0.75	0.47-1.21	0.24		1.28	0.65-2.52	0.469		0.3	0.12-0.74	0.009
Stage IV (reference: III)	2.3	1.31-4.05	0.004		1.69	1.08-2.66	0.022		1.78	0.9-3.51	0.095		3.09	1.51-6.33	0.002
Female (reference: male)	0.59	0.3-1.14	0.117		0.68	0.41-1.14	0.141		1.22	0.59-2.53	0.6		0.62	0.27-1.43	0.262
Age ≥ 50 (reference: < 50)	1.39	0.77-2.54	0.278		1.14	0.71-1.84	0.584		1.39	0.68-2.83	0.368		0.83	0.39-1.73	0.614
With comorbidity* (reference: no)	1.63	0.89-2.96	0.112		1.47	0.92-2.37	0.11		1.98	0.98-3.99	0.056		1.63	0.77-3.46	0.201

IC: induction chemotherapy + concurrent chemoradiation; CCRT: concurrent chemoradiation; HR: hazard ratio; CI: confidence interval; *comorbidity: common comorbidities include chronic diseases involving the heart, vessels, brain, lung, liver, kidneys, or the immune system and metabolism such as hypertension and diabetes.
