# Supplementary material for: Induction Chemotherapy Improved Long Term Outcomes in Stage IV Locoregional Advanced Nasopharyngeal Carcinoma
Source: Int J Med Sci. 2020 Feb 10;17(5):568–76. doi: 10.7150/ijms.42005 (PMC7085214; doi:10.7150/ijms.42005)
Supplement: Supplementary file 1 — Supplementary tables. [file ijmsv17p0568s1.pdf]

Table S1: Grade 3 or 4 adverse effects in IC + CCRT group

Chemotherapy grade 3	Chemotherapy grade 4	RT grade 3
0	0	0
0	0	Radiation mucositis: Grade 3
0	0	0
Acute pancreatitis in first IC	0	0
0	0	Grade 3 oral mucositis
Delayed vomiting	0	0
0	0	0
0	0	0
Gastric ulcers under local Bosmin injection	0	0
0	0	0
0	0	0
Delay vomiting	0	0
0	Neutropenia and activation of HCV CCRT	Neutropenia and activation of HCV
0	0	0
Delay vomiting	0	0
0	0	Oral mucositis: Grade 3 Severe pain
Hiccup grade III	Coronary artery disease	0
Hiccup grade III	0	0
Vomit (Emend used)	0	0
0	Neutropenic fever	0
0	0	0
Thrombosis subclavian vein (DVT)	0	0
0	0	0
Right arm deep vein thrombosis	0	0
0	0	0
0	0	0
0	0	0
0	0	0
Neutropenia	0	Grade 3 skin wet peeling
0	0	Paranasal sinusitis

0	0	0
Nausea with vomit	0	0
0	0	0
Vomit & Emend given	0	0
0	0	0
0	0	Radiation mucositis: grade 3
0	0	0
0	0	0
0	0	0
0	0	Hard to swallowing
Delayed nausea/vomiting	0	0
0	0	Even more sore throat
0	0	On NG tube
0	0	0
Grade 3 delayed nausea/vomiting	0	0
0	0	0
Hiccup and constipation	0	0
0	0	0
Hiccup s/p CC	0	Grade 3 oral mucositis & hiccup
Delayed Nausea/vomiting	0	0
Grade III nausea/vomiting	0	0
Chill after CC	0	Chill
0	0	Husky voice + body weight loss > 20%
0	0	0
Port-A infection cellulitis WBC down while CC	0	0
0	0	0
0	0	0
0	0	0
0	0	0
Neutropenic fever	0	0
Grade III anorexia	0	0
Severe nausea after CC	0	0
0	0	0
Severe nausea and vomiting	0	Severe nausea and vomiting

Pancytopenia (ANC:324)	0	0
0	0	0
0	Sepsis in 3rd IC from Port A	Mucositis: Grade 3
0	0	0
Acute gastroenteritis, mucositis, pneumonia, Bacteremia, Port-A infection	0	Dermatitis: Grade 3
Duodenal ulcer	Neutropenia, grade IV	0
0	0	Dermatitis: Grade 3
Delay vomiting	0	Mucositis: Grade 3 + skin reaction
Neutropenia	0	0
Hypercalcemia	Grade 4 neutropenia	Grade 3 oral mucositis
0	0	0
Leukopenia grade 3 + port-A abscess	0	0
Grade 3 nausea, anorexia on CC		Mucositis: Grade 3
0	0	0
0	0	0
0	0	0
0	0	0
0	0	0

Table S2: Grade 3 or 4 adverse effects in CCRT group

Chemotherapy Grade 3	Chemotherapy grade 4	RT grade 3
0	0	Mucositis: Grade 3
0	0	0
0	0	0
Nausea/vomiting grade 3 (vomiting 6-10/day)	0	Pharyngeal mucositis: Grade 2~3
0	0	0
0	0	0
0	0	Mucositis: Grade 3
0	0	0
0	0	Mucositis: Grade 3
0	0	Mucositis: Grade 3
0	0	Dermatitis radiation: Grade 3
0	0	Mucositis: Grade 3
0	0	0
0	0	0
0	0	Mucositis: Grade 3
0	0	0
0	0	Mucositis: Grade 3
0	0	0
0	0	0
0	0	0
Neutropenic fever on CC	0	0
0	0	0
0	0	Dermatitis: grade 3
Hypokalemia fever sepsis	0	0
0	0	Dermatitis + mucositis: Grade 3
Oral mucositis	0	Oral mucositis
Grade 3 neutropenia	0	Mucositis: Grade 3
0	0	Mucositis: Grade 3
0	0	0
0	0	0
0	0	0
0	0	0
0	0	0
0	0	0

0	0	0
0	0	0
0	0	0
0	0	0
0	0	R/T related oral mucositis
Weakness	0	Weakness
Severe fatigue and nausea	0	Mucositis Grade 3 + severe fatigue and nausea
0	0	Mucositis dermatitis: Grade 3
0	0	Dermatitis: Grade 3
0	0	0
0	0	Mucositis: Grade 3
0	0	Dermatitis + mucositis: Grade 3
Nausea: Grade 3	0	Nausea: grade 3
Nausea: Grade 3	0	0
0	0	0
0	0	Dermatitis: grade 3
Nausea/vomiting grade 3 (vomiting 6-10/day)	0	0
0	0	0
0	0	0
0	0	0
0	0	Oral mucositis: Grade 3 Severe pain
0	0	Mucositis Grade 3
0	0	0
0	0	Dermatitis: Grade 3
0	0	Dermatitis radiation: Grade 3
0	0	Dermatitis radiation: Grade 3
Neutropenia grade 3	0	Mucositis dermatitis: Grade 3
0	0	Mucositis + dermatitis grade 3
Nausea/vomiting neutropenia grade 3	0	0
Neutropenia grade 3	0	0
0	0	0
0	0	Mucositis + dermatitis grade 3
0	0	0
0	0	0
0	0	Grade 3 mucositis
	0	0

Grade 3 vomiting	0	0
0	Port-A infection after CCRT	Radiation mucositis grade 3
0	0	0
Grade 3 vomiting	0	0
0	0	0
0	0	Mucositis : grade 3
0	0	0
0	0	0
0	0	0
0	0	0
0	0	0
0	0	0
0	0	Dermatitis: Grade 3
0	0	0
0	0	Radiation dermatitis: Grade 3
0	0	Oral mucositis: Grade 3 Severe pain
0	0	0
0	0	Laryngeal/pharyngeal mucositis: Grade 3 Dermatitis radiation: Grade 3
0	0	0
0	0	Dermatitis radiation: Grade 3
0	0	Severe mucositis grade 3
0	0	Radiation mucositis: Grade 3, radiation dermatitis: Grade 3
0	0	Radiation mucositis: Grade 3
0	0	0
0	0	0
0	0	Mucositis: Grade 3 + dermatitis: Grade 3
0	0	Mucositis: Grade 3
0	0	0
Nausea/vomiting grade 3 (vomiting 6-10/day)	Nausea/vomiting grade 4 (vomiting 10+/day)	0
0	0	0
0	0	Dermatitis: Grade 3

Bacteremia fever Port-A infection	0	Severe mucositis grade 3
0	0	Aspiration pneumonia + mucositis
0	0	0
0	0	Dermatitis: Grade 3
0	0	Dermatitis + mucositis: Grade 3
0	0	0
Delayed nausea and vomit	0	Grade 3 Mucositis
0	0	Radiation mucositis grade 3
Nausea/vomiting grade 3 (vomiting ≧6/day)；	0	Dermatitis radiation: Grade 3 mucositis grade 3
0	0	Oral mucositis: grade 3
Grade 3 neutropenia	0	0
0	0	0
0	0	0
0	0	0
Delayed emesis grade III	0	Mucositis: Grade 3
0	0	0
Hold Erbitux for grade 3 mucositis	0	Grade 3 mucositis
Anorexia grade 3 Hypokalemia + neutropenia grade 3	0	0
0	0	0
0	0	0
Port-A infection s/p removal	Hypokalemia	Grade 3 mucositis
0	0	Mucositis Grade 3
0	0	0
0	0	0
0	0	0
0	0	0
0	0	0
0	0	Mucositis: Grade 3 + dermatitis: Grade 3
0	0	Radiation mucositis: grade 3
0	0	0
HB: 8.8 -> 7.7 -> 8.6	0	0
Grade 3 neutropenia	0	0
Grade 3 neutropenia	0	0

Hyponatremia vomiting with dehydration	0	0
0	0	0
0	0	0
Malaise	0	0
0	0	0
0	0	0
0	0	Mucositis: Grade 3
0	0	0
0	0	0
0	0	0
Grade 3 neutropenia nausea/vomiting grade 3 (vomiting 6-10/days)	0	Mucositis with poor intake
0	0	Dermatitis : Grade 3
0	Nausea/vomiting grade 4 (vomiting 10+/day)	Oral mucositis with dehydration
Delay vomiting	0	Mucositis: Grade 3
0	0	Dermatitis + mucositis grade 3
0	0	Oral mucositis: Grade 3 severe pain
0	0	0
0	0	Oral mucositis grade 3
0	0	Cellulitis of anterior upper neck
0	0	0
0	0	0
0	0	0

Table S3: PF dose in IC+CCRT group patient

P cycles	P dose sum (mg/m^2)	F cycles	F dose sum (mg/m^2)
3	180	3	9600
3	180	3	6000
3	180	3	6000
3	180	3	9600
3	180	3	9000
3	180	3	6000
3	180	3	6000
3	150	3	9600
2	120	2	2000
3	180	3	8000
2	120	3	5000
2	120	2	4000
2	120	2	6400
4	320	4	16000
3	180	3	6720
2	180	3	9600
3	150	3	6000
3	180	3	9600
3	180	1	2000
4	240	4	12800
3	180	3	7200
3	180	3	7200
3	180	3	6000
3	180	3	9200
3	180	3	9000
3	220	3	3000
2	120	2	2000
3	180	3	2500
2	120	0	0
3	180	0	0
0	0	3	7200
2	100	2	3200
2	70	2	3450
3	180	3	3000
4	200	4	12000

3	180	3	9600
2	100	2	4000
3	140	2	4800
3	180	3	6000
3	180	3	9600
3	180	3	9600
3	180	3	9600
3	180	3	9000
3	180	3	9600
3	180	3	9600
5	250	5	10000
3	180	3	9600
4	240	4	12800
2	120	2	6400
2	100	2	6000
4	240	4	12800
4	240	4	16000
2	120	2	8000
3	180	3	9600
3	180	3	9600
3	180	3	9600
3	180	3	9600
3	180	3	9600
3	180	3	11200
3	208	3	8000
3	180	3	9600
2	120	2	8000
4	240	4	12000
3	220	3	5200
3	180	3	9603
4	240	4	16000
3	240	3	6000
4	230	3	9600
2	120	2	8000
3	160	3	3000
3	180	3	4000
3	180	2	6400
3	240	3	9000

3	210	3	2250
One patient took only oral Tegafur for two weeks before his CCRT			
Mean of P cycle number: 2.91	Mean of cumulated P dose: 175.2 mg/m^2	Mean of F cycle number: 2.83	Mean of cumulated F dose: 7617.9 mg/m^2
	Mean of P dose per cycle: 60.3 mg/m^2		Mean of F dose per cycle: 2688.7 mg/m^2
